# A Method for the Structure-Based, Genome-Wide Analysis of Bacterial Intergenic Sequences Identifies Shared Compositional and Functional Features

**DOI:** 10.3390/genes10100834

**Published:** 2019-10-22

**Authors:** Leonardo Lenzini, Francesca Di Patti, Roberto Livi, Marco Fondi, Renato Fani, Alessio Mengoni

**Affiliations:** 1 Dipartimento di Fisica e Astronomia, Università degli Studi di Firenze, 50019 Sesto Fiorentino, Italy; francesca.dipatti@unifi.it (F.D.P.); roberto.livi@unifi.it (R.L.); 2 Istituto Nazionale di Fisica Nucleare, 50019 Sesto Fiorentino, Italy; 3 Centro Interdipartimentale per lo Studio delle Dinamiche Complesse, 50019 Sesto Fiorentino, Italy; 4 Istituto dei Sistemi Complessi, Consiglio Nazionale delle Ricerche, 50019 Sesto Fiorentino, Italy; renato.fani@unifi.it; 5 Dipartimento di Biologia, Università degli Studi di Firenze, 50019 Sesto Fiorentino, Italy; marco.fondi@unifi.it (M.F.); alessio.mengoni@unifi.it (A.M.)

**Keywords:** noncoding DNA, complex systems, clustering method, bacterial gene regulation

## Abstract

In this paper, we propose a computational strategy for performing genome-wide analyses of intergenic sequences in bacterial genomes. Following similar directions of a previous paper, where a method for genome-wide analysis of eucaryotic Intergenic sequences was proposed, here we developed a tool for implementing similar concepts in bacteria genomes. This allows us to (i) classify intergenic sequences into clusters, characterized by specific global structural features and (ii) draw possible relations with their functional features.

## 1. Introduction

It is well known that noncoding regions of DNA contain important functional elements, that mainly concern regulatory activities and changes in gene expression. Such functional elements have been identified as the participation in reproducible biochemical events, for instance Transcription Factor (TF) association, chromatin structure- or histone-modification [1]. Moreover, non-coding DNA is expected to play the role of a major substrate for critical changes, driving phenotypic modifications and differences between species or individuals, thus representing the basis for evolution as well as for disease-associated regulatory variants [2,3,4,5]. In particular, the genetic programming of complex eukaryotes appears to be significantly correlated to the variability of non-coding DNA [6,7]. Accordingly, considerable efforts have been devoted by several research groups to the study of noncoding DNA regions, mainly in eukaryotes. Traditional *in silico* approaches are based on comparative genomics, that relies upon evolutionary conservation as a property for identifying functional regions. For instance, pairwise or multiple sequence alignments have been used for predicting non-coding RNA transcripts or TF binding sites [8,9,10,11,12,13]. By comparing genomic DNA from closely and distantly related species, functional elements may be recognized on the basis of their conservation. Comparative analyses can be applied also within a species to find paralogous regions deriving from duplication events within a genome [14] or even function-related patterns based on sequence similarities [15]. These sequence-based analyses, together with experimental techniques [16,17,18], have proved quite effective for predicting functional non-coding sequences and their biological implications [19]. On the other hand, as a consequence of the variability of regulatory regions, it is quite difficult to establish the accuracy of such methods in estimating the TF binding or the transcriptional output [20,21]. In fact, it is well known that, at variance with coding sequences that are well conserved even across distantly related species, regulatory regions are relatively flexible, since most TFs tolerate considerable variations in target sequences [22]. The high turnover rate both in adjacent putatively non-functional DNA and in duplicated TF binding sites often disrupts sequence conservation and makes alignments impossible (e.g., see References [23,24,25]). Moreover, transcriptional rewiring [26] may explain events of sequence similarity loss but retention of similar function. Accordingly, in non-coding DNA, sequence similarity may not necessarily correspond to functional homology.

For all these reasons the comparative approach among specific sequence elements in the non-coding regions of DNA is certainly useful but insufficient to obtain an exhaustive description of DNA double helix functional properties. Many other approaches have been proposed to fill the gap. Among them we just mention the various techniques that run motif-finding algorithms on sets of sequences and incorporate the information of experimentally known TF binding sites in position-specific weight matrices [27,28,29] or rely on the study of the three-dimensional structure of DNA [30,31] and on neural network optimization procedures [32,33]. For instance, more recently other methods or databases aiming at identifying prokaryotic promoters have been proposed [34,35].

Along this direction, a structure-based genome-wide analysis of the eukaryotic promoters was proposed as a new approach to a comprehensive identification of the correlations between the structural properties of promoter sequences and the kind of genes they regulate [15,36]. In particular, Base composition analysis (BCA) and specific entropic indicators were employed for identifying structural similarities among different classes of promoters [37,38]. Moreover, the region around the Transcription Start Site (TSS) was shown to exhibit a very distinctive structural profile, which seems to be actively maintained by non-neutral selective constraints. Such structural profile is primarily related to a non-random distribution of nucleotides along the promoter close to the TSS [15,36]. This kind of approach has been further refined in Reference [39], where it was found that promoter sequences in *Homo sapiens*, can be classified into three main groups: two of them are distinguished by the prevalence of weak or strong nucleotides and are characterized by short compositionally biased sequences, while the most frequent regular sequences in the third group are strongly correlated with transposons. Moreover, the comparison of the promoter database of *H. sapiens* with those of other species indicates that structural complexity characterizes also the evolutionary content appearing in mammalian promoters, at variance with ancestral species in the phylogenetic tree, that exhibit a definitely lower level of differentiation among promoters. This notwithstanding, evolutionary selection of regulatory DNA sequences is at work in all organisms [40,41,42] and it is reasonable to expect that in prokaryotes also a genome-wide approach can be effective in identifying possible correlations between structure and regulation/expression of genes.

In this paper we developed a computational method based on the compositional analysis of bacterial Intergenic sequences to analyze the structure of non-coding sequences close to the TSS in various bacterial species, while searching for possible correlations with the expression, regulation and biological functions of the genes they correspond to. The overall strategy of this approach is illustrated making use of *Escherichia coli*, as a primary case study (although the method has been applied to other prokaryotes, mostly leading to overlapping results).

## 2. Materials and Methods

### 2.1. Databases

The 200-bps-long IGRs of *E. coli* have been downloaded from the National Center for Biotechnology Information (NCBI), a part of the United States National Library of Medicine (NLM), the branch of the National Institutes of Health (NIH), that houses GenBank sequence database [43], an open access that contains all publicly available nucleotide sequences and has annotated the TSC of all genes.

In order to identify the genes contained in the operons we used DOOR (*Database of prOkaryotic OpeRons*) [44,45,46], an operon database developed by Computational Systems Biology Lab (CSBL) at University of Georgia, covering 2072 bacteria genomes and with overall accuracy of 90%.

### 2.2. Shine-Dalgarno Sequences

The Shine-Dalgarno sequence (SDS) is a purine-rich ribosomal binding site, usually located a dozen bps upstream the TSC. The typical six-base consensus sequence is AGGAGG. The Base Composition Analysis (see the subsection hereafter reported in Methods) of the 200-bps-long IGSs, still including the SDSs, of *E. coli* (as well as those of the other bacteria analyzed in this paper) exhibits a peak of the density of G nucleotides in the vicinity of 10 bps upstream the TSC, thus signaling the typical occurrence of SDS in this part of these 200-bps-long IGSs, still including the SDSs. It is well known that the presence of a SDS is associated to the position of the Transcription Start Site (TSS). This indicates that the 200-bps-long, still including the SDSs, of *E. coli* contain a noncoding region that is transcribed and not translated. Since our clustering analysis (see the following section in Methods) aims at characterizing structural similarities between strictly noncoding regions, we want to eliminate from any IGS its portion, upstream the TSC, that is transcribed and not translated. Moreover, following Reference [47], we have considered as indicators of the TSS also all its subsequences: GGA, GAG, AGG, GGAG, GAGG, AGGA, AGGAG, GGAGG. In more detail, the procedure for identifying SDSs is implemented as follows: for each of the, still including the SDSs, sequences we look for the presence of a SDS in the first 25 bps upstream the TSC (The seemingly magic number 25 stems from the direct inspection of Shine-Dalgarno motifs in the 200-bps-long IGSs, still including the SDSs, of *E. coli*: the probability of finding a Shine-Dalgarbo motif upstream the TSC practically vanishes beyond 25 bps). We start looking for the longest SDS (AGGAGG) and if we do not find it we pass to shorter SDSs, proceeding in dissent order of length, up to the three bps long SDSs. When we find a sequence of nucleotides matching with a SDS we annotate the position of its first nucleotide and we associate it to the TSS of the corresponding gene or operon.

### 2.3. Spectral Clustering

The aim of the procedures described in this section is to collect the annotated IGSs into clusters depending on the similarity between the sequences. This procedure consists of three main steps:

- aligning each sequence with all the others (pairwise alignment) thus obtaining a matrix whose entries are similarity scores;
- analyzing the eigenvalues of the Laplacian matrix, computed by the similarity matrix, for determining the appropriate number of clusters;
- making use of the eigenvectors of the Laplacian matrix to work out the k-means algorithm, which allows us to associate each IGS to the selected clusters.

#### 2.3.1. Sequences Alignment

The basic idea of a sequence alignment is to identify regions of similarity that may be related with functional or structural properties as well as evolutionary relationships. Clearly, any alignment procedure cannot be based on a perfect match between sequences but it has to take into account important biological features such as mutations and insertions or deletions occurred during the evolution. For this reason, the standard approach to this problem is to implement computational methods that make use of a substitution matrix to assign positive and negative scores to nucleotide matches or mismatches and a gap penalty for matching a nucleotide in one sequence to a gap in the other one. These algorithms, in general, fall into two categories: global and local techniques. A global algorithm spans the entire length of the sequence, while a local alignment focuses on identifying regions of similarity within long sequences that are often widely different overall. In this paper we have made use of the two most popular alignment methods, the Needleman-Wunsch global algorithm [48] and the Smith-Waterman local algorithm [49] implemented in the EMBOSS package version 6.6.0 [50].

A key aspect of the procedure, which may give rise to a marked difference in the best match score calculated by the two algorithms, is the choice of the penalty value to be assigned to the introduction of a new gap in the alignment (GAPOPEN) and the value for each consecutive gap (GAPEXTEND); the scoring matrix for the nucleotide substitution has been taken equal to the standard EDNAfull matrix for both methods. Unfortunately there’s no way to set a priori the optimal choice of parameters and thus the best option is to tune the values depending on the results obtained. Regarding our work, the trials we performed suggest to use a high GAPOPEN value (typically set equal to 10) and a low GAPEXTEND penalty (0.5) in order not to penalize long gap sequences. This setting favors the scores of very similar sequences yielding an easier detection of the correct number of clusters (see section *The normalized Laplacian matrix*). Moreover, in the EMBOSS code, gaps inserted at the beginning or at the end of the sequence have no penalty. In this way, we do not observe a significant difference between the two algorithms and the outcome of aligning *N* IGSs gives the same similarity matrix *S* in both cases.

#### 2.3.2. The Normalized Laplacian Matrix

A convenient way to represent the N×N entries $s_{ij}$ of the symmetric similarity matrix *S*, is to introduce a network whose nodes coincide with the sequences, while the entry $s_{ij}$ represents the weighted link between sequence *i* and *j*. For the purpose of our work, however, dealing with a full connected network is not the best approach. The risk is that the noise induced by the fact that even the alignment of two random sequences gives a positive score, may hide the real common features among IGSs, making the clustering procedure unfruitful. For this reason, it is of paramount importance to substitute *S* with a weighted adjacency matrix *W*, for which two nodes are connected only if their alignment score is larger that a certain threshold $s^{*}$, namely $w_{ij}=s_{ij}$ if $s_{ij}⩾s^{*}$ and $w_{ij}=0$ otherwise. To estimate $s^{*}$, we have used two methods. In the first one $s^{*}$ has been estimated by reordering randomly the nucleotides of each one of the *N* annotated IGSs and then applying to this new set of N randomized sequences the alignment algorithm. The arithmetic mean of the $s_{ij}$ obtained for the randomized sequences provides a preliminary estimate of $s^{*}$. By iterating this procedure to perform a further averaging over different estimates we have checked that the preliminary estimate is pretty stable. The second method for estimating $s^{*}$ is based on the computation of the alignment score of each one of the annotated IGSs with ten realizations of the random reordering of its nucleotides. Then $s^{*}$ is computed as the arthmetic mean of the alignement scores obtained for all IGSs.

The latter method yields an estimate of the threshold that is typically higher than the one obtained with the former procedure. For instance, in Figure 7 the eigenvalues of the Laplacian matrix obtained by the second method (blue symbols) are lower than those obtained by the first method (red symbols), while their relative separation is more pronounced. Since we are interested in highlighting structural similarities between the relatively short IGSs of bacterial species, we have constructed the Laplacian matrix making use of the second method.

Finally, in order to manage a set of more homogeneous data, we have operated the normalization $w_{ij}\to w_{ij}/max\left\{w_{ij}\right\}$.

Following Reference [51], once an appropriate similarity matrix is obtained, the first step of the clustering procedure is the determination of the number of clusters. For this purpose, we introduce the normalized Laplacian $L_{sym}=D^{-1/2}\left(D-W\right)D^{-1/2}$ where the degree matrix *D* is defined as the diagonal matrix with entries $d_{i}=\sum_{j=1}^{N}w_{ij}$. In some particularly successful cases, $L_{sym}$ has a block structure and the multiplicity of its null eigenvalue determines the number of connected components. In real cases, however, data is well mixed, and $L_{sym}$ has a unique null eigenvalue corresponding to one connected component, which includes the whole data set. The solution of the problem comes from the matrix perturbation theory [52]. Indeed, given the spectrum $\lambda_{1}⩽\lambda_{2}⩽\ldots ⩽\lambda_{N}$ of $L_{sym}$, the information about the number of clusters is carried by those eigenvalues which are located close to the null one. The idea is that the actual $L_{sym}$ can be read as a perturbation of an *ideal* block matrix and thus the first *k* values of the spectrum act as fluctuations of the corresponding null eigenvector of the *ideal* case, with multiplicity *k*. In practice, the more the first *k* eigenvalues are distant from the others, the more effective will be the separation of data into the *k* groups.

#### 2.3.3. Clustering Algorithm

We are now able to apply the spectral clustering algorithm in order to assign each IGS to one of the clusters. The starting point is the computation of the first *k* eigenvectors $u_{1},\ldots ,u_{k}$ of $L_{sym}$, so as to form a new matrix $U\in R^{N\times k}$ containing the vectors $u_{1},\ldots ,u_{k}$ as columns. Let $T\in R^{N\times k}$ be the matrix obtained from *U* by normalizing the rows to norm 1, namely, $t_{i,j}=u_{i,j}/{\left(\sum_{k}u_{i,k}^{2}\right)}^{1/2}$. For i=1,…,N we denote by $y_{i}\in R^{k}$ the vector corresponding to the i-th row of *T*. The last point consists in applying the k-means algorithm to the points $y_{i}$ so as to find $A_{1},\ldots ,A_{k}$ clusters. The iterative procedure of the algorithm works as follows: first, select *k* random points as initial centroids. Then, form *k* clusters assigning each point $y_{i}$ to its closest centroid, according to Euclidean distance. Recompute the centroids as the mean of the points of each cluster. Repeat until the difference between the centroids coordinates of two consecutive steps reaches a fixed tolerance. For instance, in Figure 8 this tolerance was fixed to $10^{-8}$.

#### 2.3.4. Silhouette

Silhouette index allows to evaluate the consistency of a clustering procedure, measuring how similar an object is to its own cluster compared to other clusters. The silhouette value ranges from −1 to +1, where positive values indicate that the assignment of the object to a cluster is good, while negative values stand for a bad assignment. The clustering configuration is more appropriate the more objects are characterized by a silhouette value close to +1. Now we illustrate how to calculate the silhouette.

For a data point *i* in the cluster $C_{k}$ with $N_{k}$ elements, we define

(1) $$a\left(i\right)=\frac{1}{N_{k}}\sum_{j\in C_{k}}d\left(i,j\right),$$

where the sum is over all the data points, except for *i* and d(i,j) is the Euclidean distance between the two points. The value a(i), the average distance of *i* with all other data points in the same cluster, is as a measure of how well *i* is assigned to its cluster. Let

(2) $$b\left(i\right)=\min_{h\neq k}\frac{1}{N_{h}}\sum_{j\in C_{h}}d\left(i,j\right)$$

be the smallest mean distance of *i* to all points in any other cluster $C_{h}$ with $N_{h}$ elements, of which *i* is not a member.

The silhouette value for a data point *i* is

(3) $$s\left(i\right)=\frac{b(i)-a(i)}{\max(a(i),b(i))}.$$

So if a(i)<b(i) then s(i) will be positive, negative otherwise.

### 2.4. Base Composition Analysis

In order to characterize a set of *N* equal-length sequences, it is useful to represent the spatial distribution of each nucleotide along the IGSs by the so-called Base Composition Analysis. In practice, we compute the density $\rho_{x}\left(\ell \right)$ of each nucleotide x=A,TGC at position *ℓ* along the IGS defined as

(4) $$\rho_{x}\left(\ell \right)=\frac{1}{N}\sum_{i=1}^{N}s_{i}^{x}\left(\ell \right),$$

with $s_{i}^{x}\left(\ell \right)=1$ if in the *i*-th IGS the nucleotide *x* is present at position *ℓ*, $s_{i}^{x}\left(\ell \right)=0$ otherwise. For what concerns the annotated IGSs of the bacteria considered in this paper ℓ=−175,⋯,−1, while the position 0 corresponds to the last nucleotide of the SDS or to the TSC for those IGSs where the SDS is lacking.

**Figure 1:**
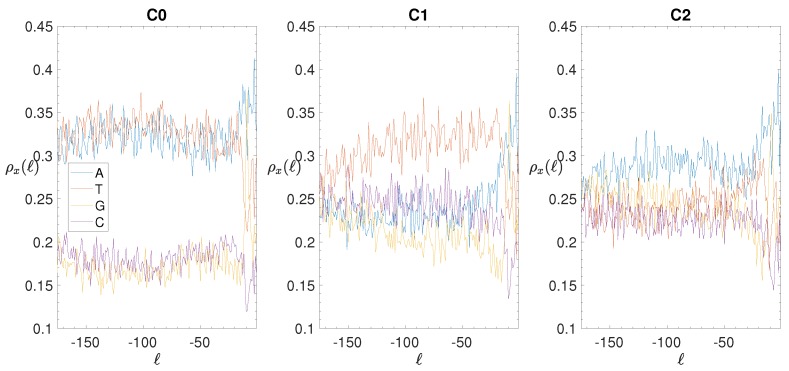
*E. coli*. Base composition analysis in the clusters of the IGSs: on the vertical axis we report the density $\rho_{x}\left(\ell \right)$ (see Section 2.4) of each of the four nucleotides x= A (blue), T(red), G (yellow), C (purple) as a function of the position *ℓ* along the IGS belonging to the clusters C0 (left panel), C1 (central panel) and C2 (right panel).

In this section, we take the opportunity to report the BCA of the three clusters obtained after the clustering procedure, as we can see in Figure 1.

Since for not too large values of *N* BCA typically exhibits sensible fluctuation, the density profile of nucleotides can be better represented by a smoothing procedure, where we proceed to a further averaging of the density inside a “window” of 2a bps, in formulae

(5) $${\bar{\rho }}_{x}\left(\ell \right)=\frac{1}{N}\sum_{i=1}^{N}\frac{1}{1+2a}\sum_{\ell^{\prime }=\ell -a}^{\ell^{\prime }=\ell +a}s_{I}^{x}\left(\ell^{\prime }\right).$$

### 2.5. About STRING

STRING (*Search Tool for the Retrieval of Interacting Genes*) is a database of known and predicted protein-protein interactions (PPI). The interactions include direct (physical) and indirect (functional) associations; they stem from computational prediction, from knowledge transfer between organisms and from interactions aggregated from other (primary) databases. Thanks to this database we can build the network of predicted associations for a particular group of genes (or proteins). The network nodes are the genes. The weighted edges represent the predicted functional associations. In fact, STRING provides a score for each protein-protein association. The scores take values from zero to one and and indicate the estimated likelihood that a given interaction is biologically meaningful, specific and reproducible, given the supporting evidence. There are seven *evidence channels* that together contribute to providing the total evidence, that is the protein-protein association score.

We have analyzed the channels separately. In particular we focused on the coexpression and cooccurrence channels.

#### 2.5.1. Coexpression

After the transcription of DNA in RNA, information is translated for the production of specific proteins. These processes are known by the collective name of gene expression. If a couple of genes exhibits consistently similar expression patterns in different experimental conditions, that means the transcription levels of the two genes rise and lower in sync, we can say that those genes are coexpressed and will receive a high association score.

Thanks to the one-to-one correspondence between one IGS and the corresponding transcriptional unit (formed by a gene or an operon), from the clustering we can build a coexpression network where the nodes are the genes and the weighted edges are given by the PPI score of the coexpression channel. We choose a threshold for the score, below which, the link is deleted, above it is arbitrarily set to one. We analyzed the characteristic properties of this undirected and unweighted graph based on gene coexpression.

#### 2.5.2. Cooccurrence

We adopted the same procedure for the cooccurrence channel. In this channel, STRING evaluates the phylogenetic distribution of orthologs of all genes in a given organism. If two genes show a high similarity in this distribution, that is their orthologs are present or absent roughly in the same subsets of organism, then a high score of the cooccurence channel is assigned.

### 2.6. COG Categories Enrichment

To conduct functional enrichment, each gene whose upstream intergenic region was clustered in one of the three clusters was assigned to a specific functional category using a BLAST [53] search against the COG database [54], with default parameters and considering a hit as significant if E-value $<1e^{-20}$. The exact binomial test implemented in the R package [55] was used to assess over- and under-represented functional categories against the corresponding genomic background. The Blast2Go package [56] was used to assess over- and under-represented GO terms in each cluster.

## 3. Results and Discussion

### 3.1. Identification and Clustering of Intergenic Sequences

**Figure 2:**
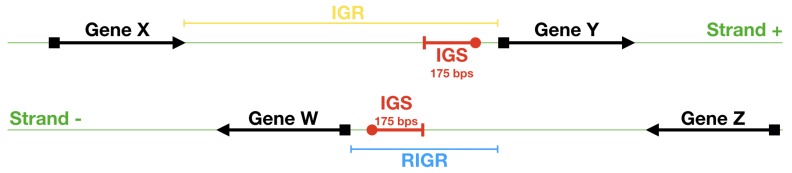
Scheme of identification of IGR, RIGR and IGS in DNA bacterial strands: in green we denote the two DNA strands; the arrows denote the transcription direction; the black squares locate the TSC, while the red circles locate the TSS.

**Figure 3:**
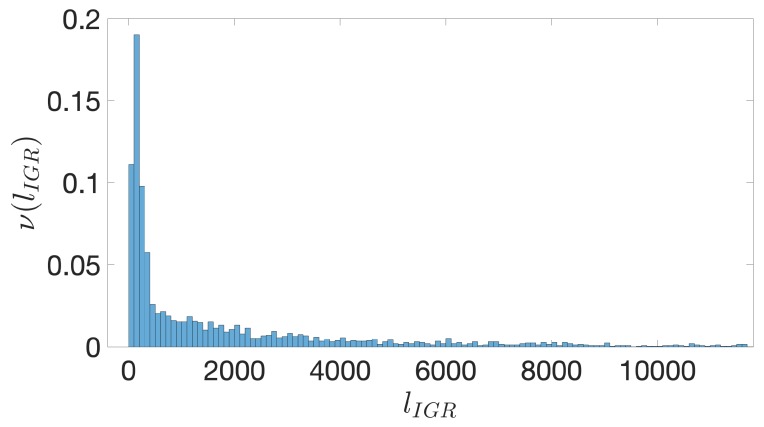
*E. coli*. The frequency $\nu (l_{IGR})$ of IGRs versus their length, $l_{IGR}$, expressed in bps. The binning is over 100 bps. The distribution is truncated at 12,000 bps.

In analogy with what studied in the previous papers concerning the study of structural features of promoters in eukaryotes, we expect that noncoding regions of bacterial DNA close to the TSS are correlated with regulation and expression of genes. In what follows we call these regions intergenic sequences (IGSs). In order to identify the IGSs we have to introduce first the concept of intergenic region (IGR): this region extends between the Translation Start Codon (TSC) of one gene and the end of the previous coding region on the same strand (see Figure 2). For instance in *E. coli* the average length of the IGRs is close to 2000 base pairs (bps) on both strands (see Figure 3). Data employed to produce this figure have been dowloaded from NCBI (National Center for Biotechnology Information) (To avoid potential background noise, in this work we considered only chromosomal DNA and discarded plasmid DNA) [43]. This database provides also the position of TSC, identified by an ATG triplet: in Figure 2 the TSC is represented by a black square.

**Figure 4:**
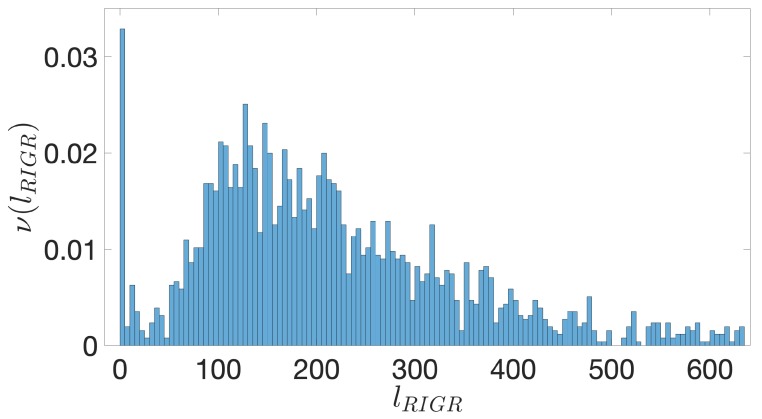
*E. coli*. The frequency $\nu (l_{RIGR})$ of restricted intergenic regions (RIGRs) versus their length, $l_{RIGR}$, expressed in bps. The binning is over 5 bps and the distribution is truncated at 600 bps. The peak close to 0 is due to the simplifying assumption of setting to 0 the contribution from overlapping coding regions.

It is well known that the genome of bacterial species exhibit quite peculiar structures. For instance, they contain operons, that is, groups of genes separated by short noncoding regions that we assumed to be poorly relevant to our analysis. Accordingly, we have associated to the entire group of genes inside the operons a single IGR upstream the first TSC. Moreover, most of these IGRs contain reverse complements of other genes on the opposite strand. A first step in the direction of the identification of IGSs amounts to restrict the extension of IGRs to the regions between genes, irrespectively on the strand they belong to. We term these regions restricted IGR (RIGR) (see Figure 2). The outcome of this procedure is a collection of RIGRs with different lengths whose distribution is reported in Figure 4. Since the statistical methods that we are going to use in the following sections necessitate equal-length sequences, we have fixed this length by adopting the following criterion: we have evaluated the average length of RIGRs, which has resulted to be approximately 250 bps and, in order to increase the statistical significance, we have subtracted from this length the variance of the corresponding distribution, which amounts to about 50 bps, thus yielding sequences of length of 200 bps upstream the TSC.

**Figure 5:**
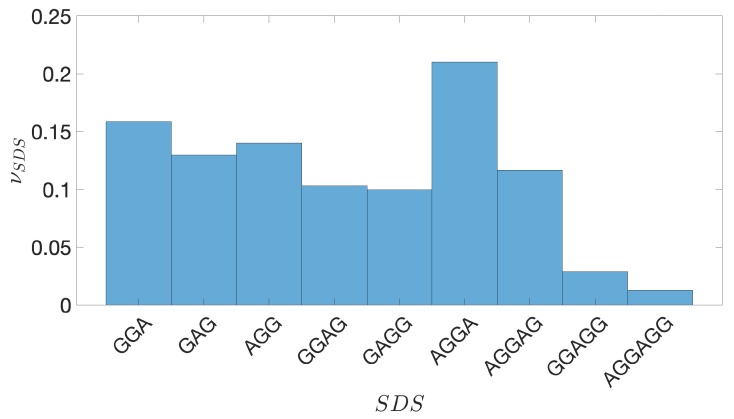
*E. coli*. The frequency $\nu_{SDS}$ of the different Shine-Dalgarno sequences (SDSs) located upstream the Translation Start Codon (TSC), listed along the horizontal axis.

**Figure 6:**
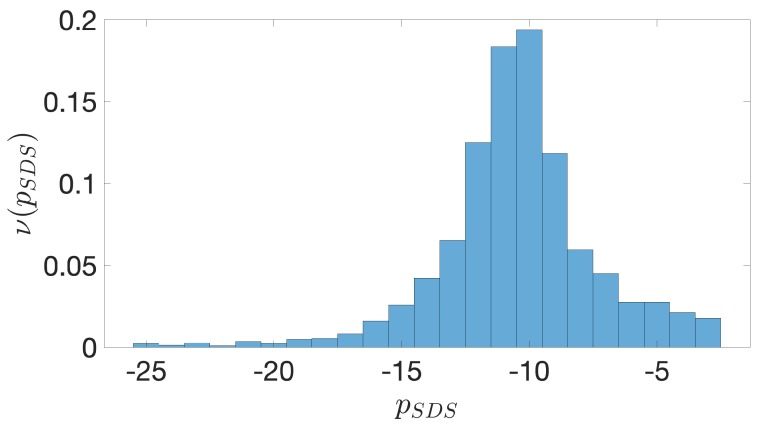
*E. coli*. The frequency $\nu (p_{SDS})$ of the position $p_{SDS}$ of the SDSs upstream the TSC.

As a final step for the identification of the equal-length IGSs, we have considered that regulatory features should be better ascribed to structural patterns belonging to sequence upstream of the Transcription Start Site (TSS) (this is denoted by a red circle in Figure 2). We assume that the position of TSS in bacterial genomes corresponds to the first nucleotide of a Shine-Dalgarno sequence (SDS), everywhere this sequence is found. For instance, in *E. coli* the SDSs are found in approximately 88% of the previously identified sequences, extending over 200 bps upstream the TSC. The different kinds of SDSs and their frequency in *E. coli* genome are reported in Figure 5. By performing a complete identification of SDSs in *E. coli* genome, we have checked that they are typically found in a range extending over the first 25 bps upstream the TSC (see Figure 6). Accordingly, we adopt the criterion of considering as IGSs those sequences extending upstream 175 bps from the TSS (In order to remove the ambiguity due to the possible presence of various SDSs in the IGR upstream the TSC we have adopted the criterion of taking the longest one as a reference for identifying the corresponding IGS) (see Figure 2). For those genes that are not preceded by a SDS, the TSS coincides with the TSC and consistently we identify the IGS with the 175 bps upstream the TSC.

The overall selection procedure applied to the *E. coli* NCBI database provides us 2553 IGSs, each one made of 175 bps. The same criterion has been adopted for the other bacterial species (see Appendix A) and we have found that a length of 175 bps for equal-length IGSs applies also to the other species.

It is evident that the criterion adopted for identifying equal-length IGSs unavoidably introduces portion of coding or reverse complement of coding sequences into the statistical sample of IGSs. On the other hand we have directly checked that *E. coli* only 434 IGSs contain more than 50% of coding portions and this poorly affects the statistical significance of the chosen sample. Similar figures are found for the other bacterial species analyzed in this paper.

**Figure 7:**
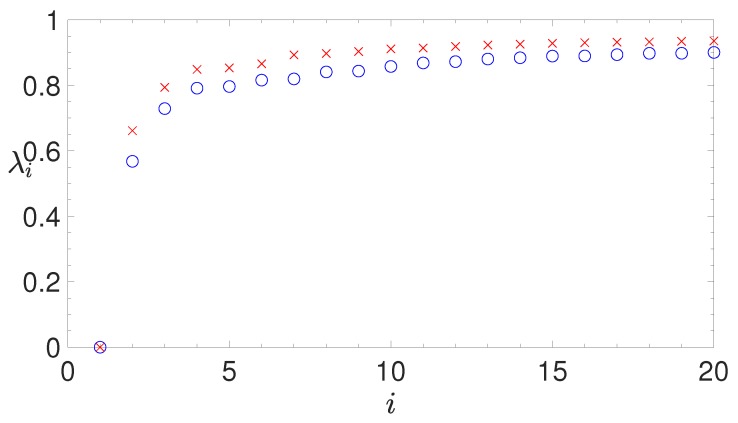
*E. coli*. The first twenty eigenvalues in ascending order of the normalized Laplacian matrix obtained by the alignment of the intergenic sequences (IGSs). Red crosses and blue circles correspond to different values of the similarity threshold, determined by the two statistical approaches described in Section 2.3.2. A better discrimination of the three main eigenvalues is obtained for a higher similarity threshold (blue circles), which corresponds to the second statistical approach, better suited for short sequences, as the annotated IGSs.

Then, these IGSs have been analyzed using a global alignment algorithm implemented by EMBOSS (version 6.6.0). The details of this analysis are described in section *Alignment Algorithm* in Methods. The following step is the application of the same clustering strategy adopted for *H. sapiens* in Reference [39], that takes into account the global properties of the identified IGSs instead of specific short regulatory motifs. The clustering procedure described in section *Spectral Clustering* in Methods is based on the spectral analysis of a similarity matrix: the entries of such matrix are obtained by the alignment algorithm that quantifies the similarity between IGSs. Since the number of identified IGSs in *E. coli* is relatively small, the alignment protocol and the diagonalization of the similarity matrix can be performed avoiding the computational limitations encountered for much larger sets of promoters, as those typically found in eukaryotes (see Reference [39]). As described in Section *The Normalized Laplacian Matrix* in Methods, the eigenvalues of the Laplacian matrix, associated to the similarity matrix, are expected to highlight the presence of possible clusters of IGSs for *E. coli*. The result of our analysis is shown in Figure 7, where we report these eigenvalues in ascending order. Symbols with different colors correspond to the eigenvalues obtained for two different values of the similarity threshold (see Section *The Normalized Laplacian Matrix* in Methods).

In particular, the red eigenvalues have been obtained by the unbiased averaging procedure adopted for estimating the similarity threshold in eukaryotes [39] (this is the first method described in Section 2.3.2). Since in *E. coli* the length of IGSs is definitely smaller than the one of eukaryotic promoters, we have adopted a more effective statistical procedure for the determination of the similarity threshold, which actually yields a better discrimination of the eigenvalues (this is the second method described in Section 2.3.2). By this procedure we have obtained the blue eigenvalues shown in Figure 7. They allow us to identify three different clusters, corresponding to the three lowest nonzero eigenvalues, that can be distinguished from the total set, because of their sensibly different values. Hereinafter they will be referred to as C0, C1 and C2.

**Figure 8:**
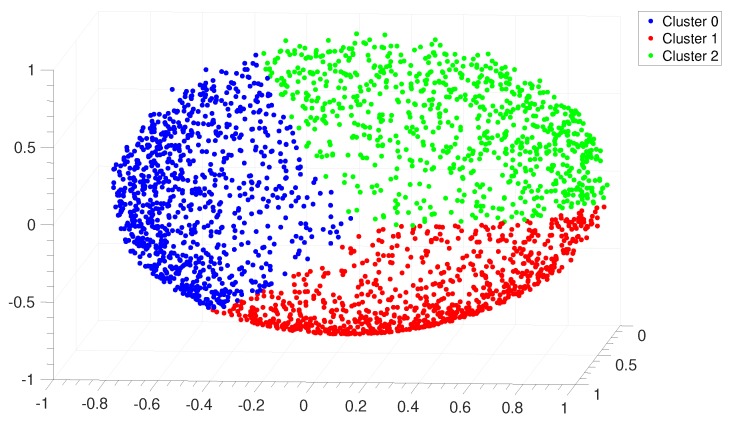
*E. coli*. Distribution of points in the clustering space relative to the alignment of the IGSs in *E. coli*. Each point represents an IGS and the color code corresponds to the three clusters identified by the *Clustering Algorithm* described in Section 2.3.3.

The reliability of this procedure is illustrated by representing the distribution of IGS in the so-called clustering space, shown in Figure 8. Each point in this space corresponds to an IGS, while IGSs with a high similarity score are represented as nearby points. Each of the 2553 IGS has been unambiguously associated to one of the three clusters by applying the metric criterion described in Section 2.3.3.

**Figure 9:**
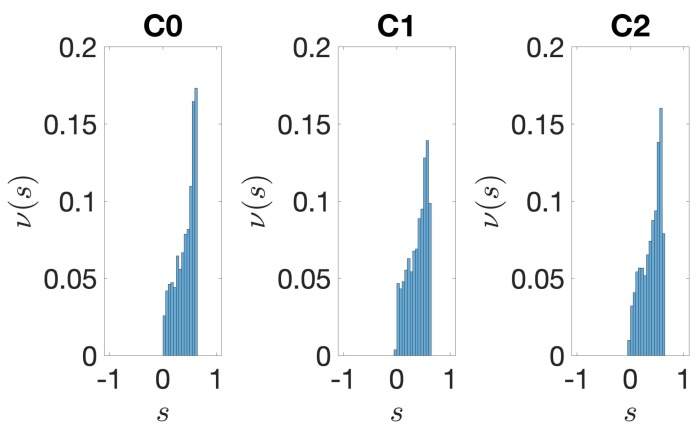
*E. coli*. Distribution of silhouette values relative to the clustering of the IGSs. On the vertical axis we report the frequency ν(s) of IGSs versus the silhouette value *s*; this value is between −1 and +1. The average values are 0.42 for cluster C0, 0.39 for C1 and 0.39 for C2.

To ascertain the consistency of the clustering procedure we have calculated the silhouette values of each point, calculated with the Euclidean distance in the clustering space (see Section 2.3.4). The distributions of these values for each cluster are reported in Figure 9. We can observe that the vast majority of values are positive, with a shift of the distribution towards the value +1, thus confirming that the clustering configuration is appropriate (We have observed that the silhouette criterion improves for the division into two clusters corresponding to the first two eigenvalues of the Laplacian matrix). On the other hand the heuristic rule to establish the number of appropriate clusters (see Section 2.3.2) amounts to choose it for first eigenvalues which maintain a significant difference between each other. This is why we have chosen to consider three clusters. A posteriori this heuristic choice is justified by the significantly different structural features characterizing the BCA of the three clusters, as shown in Figure 11).

The clustering method has been applied also to bacterial species different from *E. coli*, a Gram positive bacterium (*Bacillus subtilis*) and an extremophilic bacterium (*Pseudoalteromonas haloplanktis*). The results are reported in Appendix A.

Altogether, the analysis based on clustering by alignment yields similar results for IGSs in different bacterial species. This indicates that, irrespectively of the considered bacterium, each identified cluster of IGSs is associated to the presence of global structural properties. Now, the main question concerns the identification of the structural features characterizing the different clusters.

### 3.2. Structural Features of Clusters

**Figure 10:**
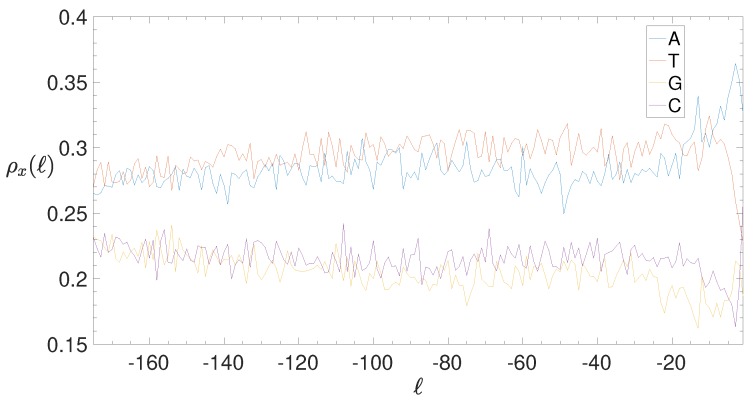
*E. coli*. Base composition analysis (BCA) of the IGSs: on the vertical axis we report the density $\rho_{x}\left(\ell \right)$ (see Section 2.4) of each of the four nucleotides x= A (blue), T(red), G (yellow), C (purple) as a function of the position *ℓ* along the annotated 2553 IGSs.

The complex structure of nucleotide sequences in the IGSs considered in this paper is essentially due to the presence of some regular patterns, that allows for a structural clustering. For instance, although obviously regulatory motifs exist also in bacteria, they are much less complex (typically, homogeneous sequences) and much shorter than in eukaryotes [20,57,58]. In Figure 10 we report the BCA of all the 2553 IGSs of *E. coli*: it has been obtained by measuring the positional density of nucleotides along both strands. The first feature that emerges is the well-known dominance of weak nucleotides (A and T) with respect to strong ones (G and C). Only close to the null position one observes peculiar peaks, corresponding to the typical enrichment of purines close to the TSS. This is an indication that our selection procedure of IGSs consistently identifies such known enrichment [59].

**Figure 11:**
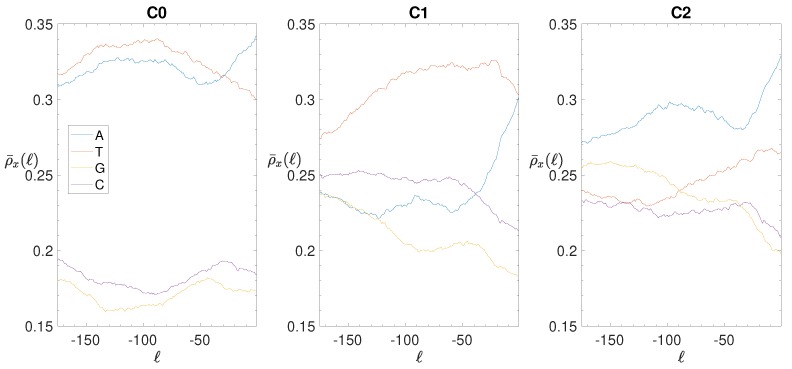
*E. coli*. Smoothed Base composition analysis in the clusters of the IGSs: on the vertical axis we report the averaged density $\bar{\rho }\left(\ell \right)$ for a=15 bps (see Section 2.4) of each of the four nucleotides A (blue), T(red), G (yellow), C (purple) as a function of the position *ℓ* along the IGSs belonging to the clusters C0 (left panel), C1 (central panel) and C2 (right panel).

If one subdivides the IGSs into the three clusters represented in the clustering space shown in Figure 8, one obtains the smoothed BCA (see (5) in Section 2.4, where is reported also the original BCA) reported in the panels of Figure 11. Cluster C0 is quite similar to the total BCA, although the separation between weak and strong nucleotides is amplified. Cluster C1 is characterized by the dominance of T nucleotides, the depression of G nucleotides, while A and C nucleotides exhibit a similar intermediate dependence on the position. Finally, Cluster C2 shows similar trends with respect to C1, with weak and strong nucleotides exchanging their role between themselves. *A posteriori* we can conclude that the clustering procedure is effective in identifying differences and similarities among the annotated IGSs. Anyway, at variance with eukaryotes, the noncoding regions of bacterial species exhibit a definitely lower level of complexity.

A more careful inspection of the BCA analysis of *E. coli* indicates that the structural differences among the three clusters is associated to the presence of regular motifs of weak nucleotides, like homogeneous patches of A and T or period-2 sequences made of AT pairs. In fact, we have found that C1 and C2 contain IGS’s that are typically enriched by homogeneous segments of T and A nucleotides, respectively. These segments extend over a few to some tens of nucleotides, while their most frequent length (as observed also in eukaryotes [39]) is close to six nucleotides. For instance, the number of homogeneous T-segments of length equal or larger than 6 nucleotides in the IGSs of C1 is approximately four times larger than homogeneous A-segments and AT-segments. Similarly, in C2 homogeneous A-segments occur twice with respect to homogeneous T-segments and five times more than AT-segments. Conversely, in C0 there are more regular A-, T- and AT-segments than those found in the other clusters and their absolute numbers are comparable (238, 292 and 185, respectively). Actually, we have also found that there is a sort of symmetry between the IGSs in C1 andC2, where homogeneous segments of weak nucleotides of the IGS in one strand appear as reverse complements in another IGS on the opposite strand. Such homogenous motifs have been recognized as typical sequences, favoring the diffusion of transcription factors along the DNA chain in search of the TSS [60].

Anyway, we are aware that the annotated IGSs of *E. coli* contain coding portions. More precisely, only 1356 IGSs do not contain any coding portion and half of them are found to belong to C0, while the remaining IGSs are approximately equally shared between C1 and C2. The average length of noncoding portions in the remaining 1197 IGSs is 102 bps and again they are almost equally shared in C1 and C2, while only 262 are contained in C0. This figures indicate that our clustering analysis is certainly influenced by the presence of coding portions, despite they play a minor role with respect to noncoding ones. On the other hand, there is not a sharp correspondence between the content of coding portions and the IGSs contained in the three clusters and we can conclude that the statistical significance of our clustering analysis is sufficient for identifying structural differences and similarities among the annotated IGSs.

**Figure 12:**
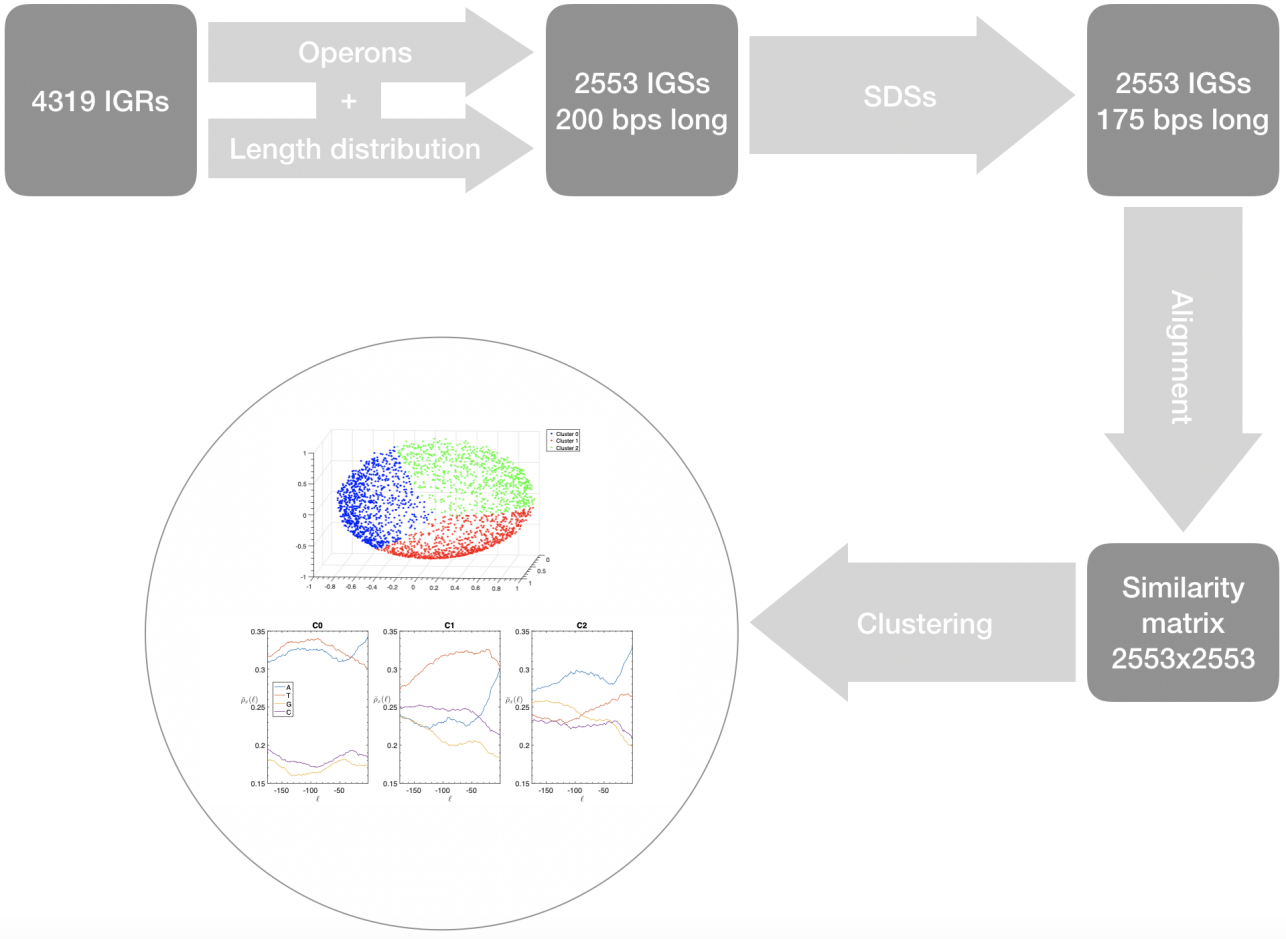
Workflow of the clustering procedure described in this section.

In summary, the proposed clustering method, summarized in Figure 12, allows us to detect specific similarities among IGSs associated also to relatively short regular subsequences. As shown in Appendix A these features are conserved in other bacterial species.

### 3.3. Correlations between Clustering and Biological Features

Once grouped IGSs into clusters the further step was to investigate possible correlations inside each cluster with biological properties. This task has been accomplished making use of the STRING database [61], which provides us information about various features related to the interactions in genetic networks. In particular, we have focused our analysis on genetic co-expression and co-occurrence in *E. coli*: details about the content of biological information associated to such features and the way they are quantified by a score is shortly discussed in section *About STRING* in Methods.

We have considered all genes and operons associated with the IGSs belonging to a cluster and we have constructed the corresponding genetic network, whose nodes represent single genes as well as genes belonging to an operon (we indicate with $N_{\mathrm{genes}}$ the total number of nodes of the network and with $N_{\mathrm{IGS}}$ the number of IGSs in a cluster). A network link is established between two genes if the corresponding element in the matrix determined by the score of the STRING algorithm overtakes a threshold value, that we have fixed to 700, in order to obtain a sparse matrix with a high level of “affinity” between pairs of connected nodes (see section *About STING* in Methods). Then, we have computed the dimension, $N_{\mathrm{LCC}}$, and the total numbers of links per node, $N_{\mathrm{link}}$, of the largest connected component (LCC) of the network. The results are reported in Table 1 and Table 2, together with the average values (${\bar{N}}_{\mathrm{LCC}}$ and ${\bar{N}}_{\mathrm{link}}$) and the variances ($\sigma_{\mathrm{LCC}}$ and $\sigma_{\mathrm{link}}$) of the same quantities, obtained by averaging over a 1000 random samplings of the IGSs (and of the corresponding genes) in the networks, built up by grouping the same total number of IGSs in each cluster. The values obtained by our clustering method correspond to values of the co-expression and co-occurrence indicators, that are typically close to, or just beyond, the border of the variance range.

Hence, clustering IGSs by structural similarity suggests the existence of a correlation with co-expression and co-occurrence. Establishing more precise relations, if any, with specific motifs appearing in the IGS’s belonging to each cluster demands a deeper inspection about the mechanisms associated to gene expression and regulation. However, this issue is beyond the aims of this paper.

**Figure 13:**
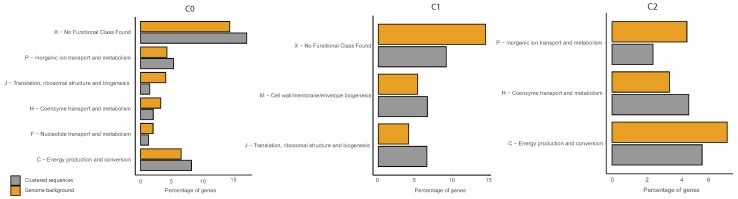
COG functional enrichment analysis of clustered genes in *E. coli*. We report the significantly enriched or depleted COG functional categories belonging to each of the identified clusters (C0, C1, C2) in respect to the genome background. *Clustered Sequences* refers to the functional annotation of the sequences that were clustered according to our method (i.e., after the analysis of IGSs) in each of the three clusters. *Genome background* refers to the functional annotation of the entire genome (i.e., of each gene of the considered organism).

**Figure A11:**
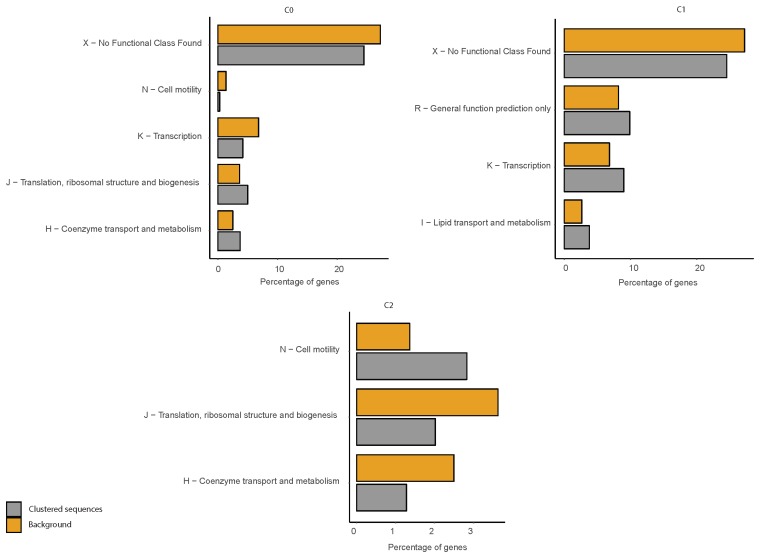
COG functional enrichment analysis of clustered genes in *B. subtilis*. We report the significantly enriched or depleted COG functional categories belonging to each of the identified clusters (C0, C1, C2) in respect to the genome background. *Clustered Sequences* refers to the functional annotation of the sequences that were clustered according to our method (i.e., after the analysis of IGSs) in each of the three clusters. *Genome background* refers to the functional annotation of the entire genome (i.e., of each gene of the organism considered).

**Figure A22:**
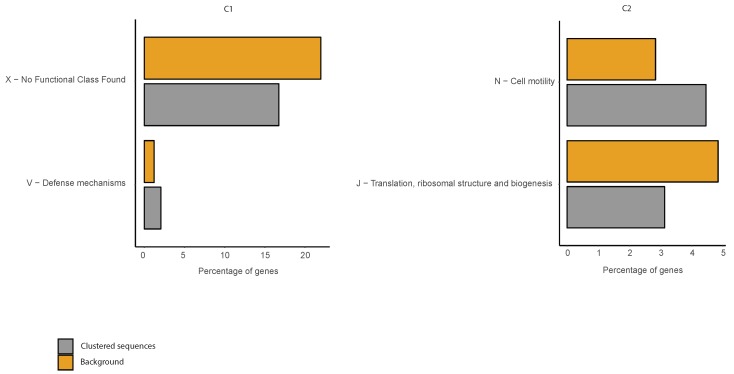
COG functional enrichment analysis of clustered genes in *P. haloplanktis*. We report the significantly enriched or depleted COG functional categories belonging to each of the identified clusters (C0, C1, C2) in respect to the genome background. *Clustered Sequences* refers to the functional annotation of the sequences that were clustered according to our method (i.e., after the analysis of IGSs) in each of the three clusters. *Genome background* refers to the functional annotation of the entire genome (i.e., of each gene of the organism considered).

In order to understand whether genes belonging to a specific biological function were over-represented in any of the identified clusters, we performed a functional enrichment analysis using COG categories and evaluating statistical significance (if any) using a negative binomial test. Data obtained for *E. coli* are shown in Figure 13, whereas results for *B. subtilis* and *P. haloplanktis* are reported in Supporting Information (see Figure A11 and Figure A22).

Overall we observed a few enriched functional categories for each of the *E. coli* clusters. In particular, 3 COG functional categories were found to be over- and down-represented in C0, respectively. The first set included genes involved in the transport and metabolism of inorganic ion, in the production and conversion of energy as well as genes lacking a functional annotation. The second set included genes involved in information processing (translation, ribosomal structure and biogenesis), coenzyme and nucleotide metabolism. The other two clusters included over-represented categories (Cell membrane biogenesis and metabolism and information processing of C1 and coenzyme metabolism of C2) as well as down-represented ones (inorganic ion transport and energy production and metabolism of C2). Despite only a few cellular processes displayed a significant trend in the clustering of IGSs, it is worth noticing that some functional categories fall in more than one cluster but always with an opposite trend according to the functional enrichment analysis. For instance, this is the case of COG J over-represented in C1 and down-represented in C0, COG P and C, over-represented in C0 and down-represented in C2 and COG H over-represented in C2 and down-represented in C0. It might be tempting to speculate that this peculiar distribution of genes belonging to the same process category among the identified clusters and due to the structure of their IGSs could reflect differences in the regulatory features of the corresponding genes. Additional analyses/experiments will be needed to evaluate the robustness of this association.

## 4. Conclusions

In this paper we report a method shown and summarized in the workflow of Figure 12, that allowed us to classify into groups the IGSs of a bacterial genome. This method can be sketched in five steps.

1. First we identify the IGRs, all the noncoding portions that are upstream the TSCs annotated in the genome considered, including also the reverse complement of the genes on the opposite strand.
2. Starting from the IGRs we can build the set of the IGSs by selecting only the noncoding part between the TSC of a gene and the end of the previous one, regardless of the strand where is located. With the help of an operon database only those that precede the TSC of a transcriptional unit (single gene or operon) are selected. We annotate the length in term of bps of each IGS. Calculating the distribution of the lengths, mean and standard deviation, we can choose a common length for all the IGSs.
3. The presence of the SDSs is useful to detect approximately the position of the TSSs so as to eliminate the transcripted and not translated part for each IGS.
4. These “cleaned up” noncoding sequences can be compared using alignment algorithms that provide a similarity score between them.
5. Similarity matrix containing these scores is processed with a clustering algorithm and the IGSs are divided into clusters based on compositional similarities.

Finally, it is possible “to interweave” the information contained in each cluster with the ones associated with biological-type databases in order to check if they are expression of functional characteristics.

Now that we have illustrated our method for classifying IGSs a priori on the basis of structural properties and a posteriori on the one of biological functionalities, it makes sense to compare it with other similar methods, in particular the ones already mentioned in References [8,9,10,11,12,13], to highlight the differences. In [8,9,10] non-coding conserved sequences are taken into consideration. In Reference [8], it is shown that conserved non-coding segments contain an enrichment of transcription factor binding sites, when compared to the sequence background in which the conserved segments are located and that this enrichment of binding sites was not observed in coding sequence. Also the comparative sequence analysis executed in [9] for identifying sequences that are conserved across multiple species revealed substantial fraction of the bases within this sequences (approximately 70%) resides within non-coding regions. Initial characterization of these “Multi-species Conserved Sequences” has revealed sequences that correspond to clusters of transcription factor-binding sites, non-coding RNA transcripts and other candidate functional elements. In Reference [10] it is found that conserved non coding sequences are significantly more conserved than protein-coding genes and noncoding RNAS (ncRNAs) within the mammalian class, from primates to monotremes to marsupials. The pattern of substitutions in conserved non coding sequences differed from that seen in protein-coding and non coding RNA genes and resembled that of protein-binding regions. A three-way multiple alignment between the genomes (human, mouse and rat) carried on in Reference [11] to detect non coding sequences is at the base of a graph theoretic clustering algorithm, akin to the highly successful methods used in elucidating protein sequence family relationships. The algorithm is applied to a highly filtered set of about 700,000 human-rodent evolutionarily conserved regions, not resembling any known coding sequence. From these, roughly 12,000 non-singleton clusters have been obtained, dense in significant sequence similarities. Reference [12] contains a method that can accurately identify pairs of functional noncoding orthologs at evolutionarily diverged loci by searching for conserved transcription factor binding sites arrangements, detecting approximately 300 pairs of diverged elements that are likely to share common ancestry and have similar regulatory activity. It is argued that transcription factor binding sites composition is often necessary to retain and sufficient to predict regulatory function in the absence of overt sequence conservation, revealing an entire class of functionally conserved, evolutionarily diverged regulatory elements. In Reference [13] a comparative method for genome-wide identification of families of regulatory RNA structures had been proposed: it has been applied to a 41-way genomic vertebrate alignment in order to find regulatory RNA structures that are often members of families with multiple paralogous instances across the genome. Family members share functional and structural properties, which allow them to be studied as a whole, facilitating both bio-informatic and experimental characterization. Known families identified include both noncoding RNAs and cis-regulatory structures. They also identify tens of new families supported by strong evolutionary evidence and other statistical evidence, such as GO term enrichments. These findings exemplify the diversity of post-transcriptional regulation and provide a resource for further characterization of new regulatory mechanisms and families of noncoding RNAs.

In the light of the methods described above, ours differs significantly from all of them, because it aims at the identification of structural elements or properties inherent the whole set of IGSs inside a species and, then, at a comparison among different species. In particular, the three main identifying features of our method are listed hereafter:

1. the object of our research, the IGSs, are sequences of DNA upstream the TSS, charged with regulation at its very first step, since it is non-coding non-transcribed DNA (unlike RNA non-coding);
2. IGSs belonging to the same organism are considered and the structural similarities are identified between sequences upstream the TSS unambiguously determined by the identification procedure, regardless of whether they are conserved or not;
3. The IGSs are 175-bps long and the alignment procedure takes into consideration the whole sequence globally in its length without focusing specifically on the transcription factor binding sites allowing a correspondence between functional properties and large-scale structural features.

## Figures and Tables

**Table 1:** Coexpression networks for *E. coli*. We compare the features of the coexpression networks for each cluster between the three clusters obtained by the clustering method and other three clusters obtained by averaging over a 1000 random samplings of the IGSs.

	$N_{\mathrm{IGS}}$	$N_{\mathrm{genes}}$	$N_{\mathrm{LCC}}$	${\bar{N}}_{\mathrm{LCC}}$	$\sigma_{\mathrm{LCC}}$	$\frac{N_{\mathrm{LCC}}-{\bar{N}}_{\mathrm{LCC}}}{\sigma_{\mathrm{LCC}}}$	$N_{\mathrm{link}}$	${\bar{N}}_{\mathrm{link}}$	$\sigma_{\mathrm{link}}$	$\frac{N_{\mathrm{link}}-{\bar{N}}_{\mathrm{link}}}{\sigma_{\mathrm{link}}}$
C0	930	1543	32	53.6	21.4	−1.01	82	341.9	238.8	−1.09
C1	812	1451	62	43.3	17.6	1.06	707	269.4	194.0	2.26
C2	811	1325	59	42.7	17.8	0.92	261	263.8	197.2	−0.01

**Table 2:** Cooccurrence networks for *E. coli*. We compare the features of the cooccurrence networks for each cluster between the three clusters obtained by the clustering method and other three clusters obtained by averaging over a 1000 random samplings of the IGSs.

	$N_{\mathrm{IGS}}$	$N_{\mathrm{genes}}$	$N_{\mathrm{LCC}}$	${\bar{N}}_{\mathrm{LCC}}$	$\sigma_{\mathrm{LCC}}$	$\frac{N_{\mathrm{LCC}}-{\bar{N}}_{\mathrm{LCC}}}{\sigma_{\mathrm{LCC}}}$	$N_{\mathrm{link}}$	${\bar{N}}_{\mathrm{link}}$	$\sigma_{\mathrm{link}}$	$\frac{N_{\mathrm{link}}-{\bar{N}}_{\mathrm{link}}}{\sigma_{\mathrm{link}}}$
C0	930	1543	338	267.7	35.4	1.99	1022	665.5	123.2	2.89
C1	812	1451	146	214.4	35.4	−1.93	326	503.8	110.5	−1.61
C2	811	1325	179	213.8	35.2	−0.99	447	497.1	111.1	−0.45

**Figure A1:**
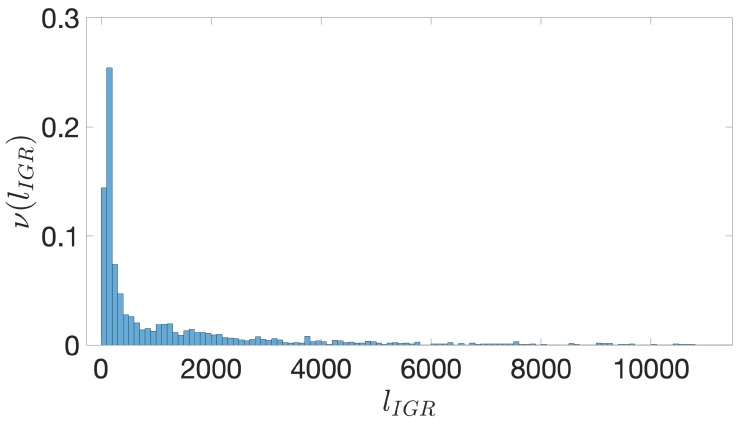
*B. subtilis*. The frequency $\nu (l_{IGR})$ of IGRs versus their length, $l_{IGR}$, expressed in bps. The binning is over 100 bps. The distribution is truncated at 12,000 bps.

**Figure A2:**
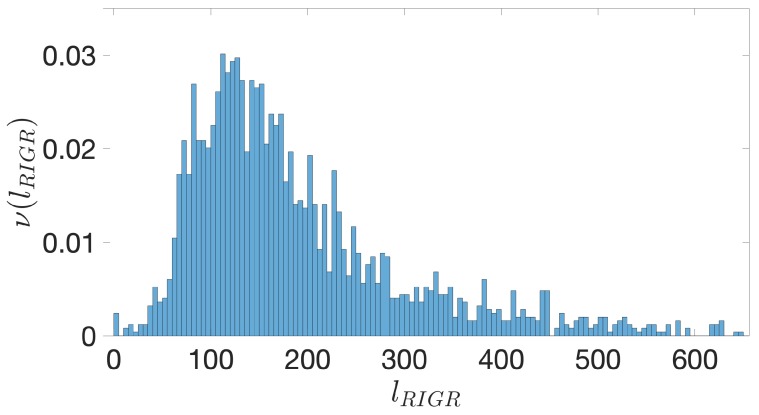
*B. subtilis*. The frequency $\nu (l_{RIGR})$ of RIGRs versus their length, $l_{RIGR}$, expressed in bps. The binning is over 5 bps and the distribution is truncated at 600 bps. The peak close to 0 is due to the simplifying assumption of setting to 0 the contribution from overlapping coding regions.

**Figure A3:**
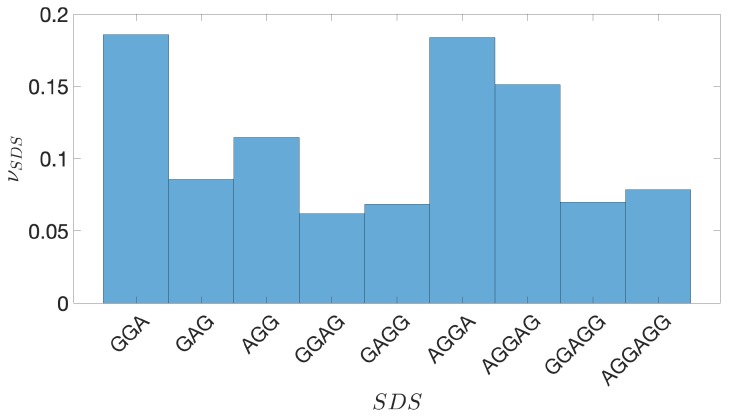
*B. subtilis*. The frequency $\nu_{SDS}$ of the different SDSs located upstream the TSC, listed along the horizontal axis.

**Figure A4:**
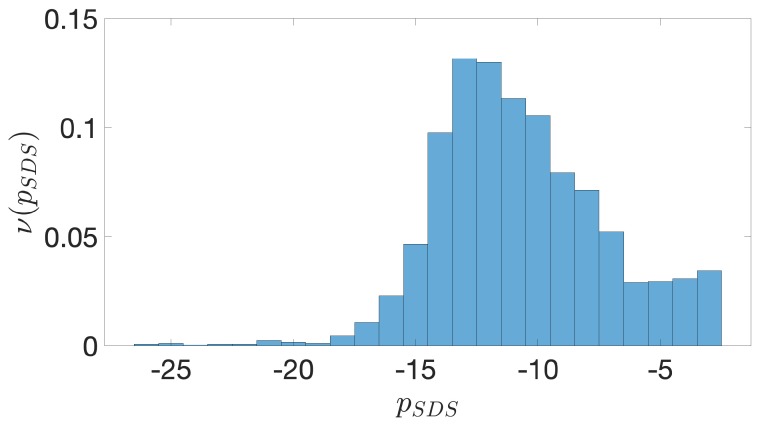
*B. subtilis*. The frequency $\nu (p_{SDS})$ of the position $p_{SDS}$ of the SDSs upstream the TSC.

**Figure A5:**
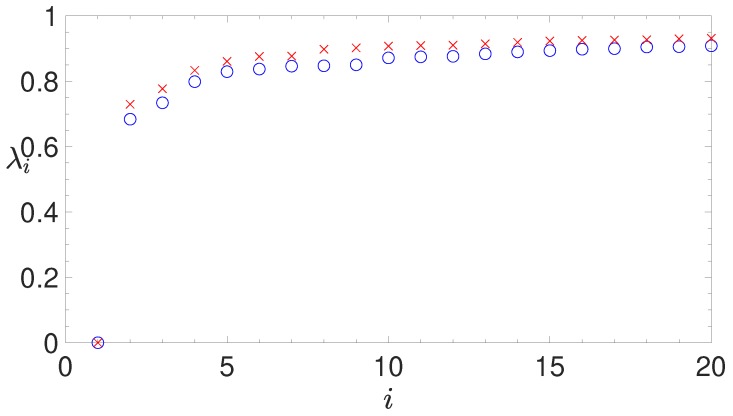
*B. subtilis*. The first twenty eigenvalues in ascending order of the normalized Laplacian matrix obtained by the alignment of the IGSs. Red crosses and blue circles correspond to different values of the similarity threshold, determined by the two statistical approaches described in Section 2.3.2. A better discrimination of the three main eigenvalues is obtained for a higher similarity threshold (blue crosses), which corresponds to the second statistical approach, better suited for short sequences, as the annotated IGSs.

**Figure A6:**
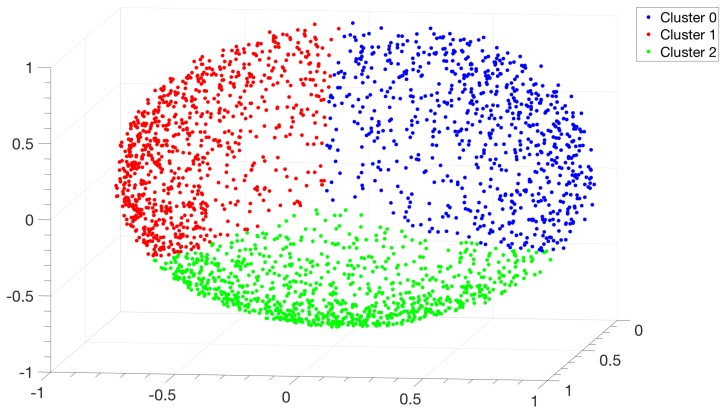
*B. subtilis*. Distribution of points in the clustering space relative to the alignment of the IGSs. Each point represents an IGS and the color code corresponds to the three clusters identified by the *Clustering Algorithm* described in Section 2.3.3.

**Figure A7:**
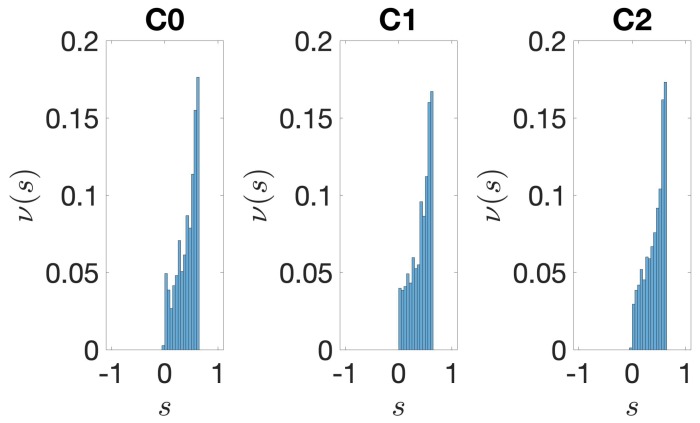
*B. subtilis*. Distribution of silhouette values relative to the clustering of the IGSs. On the vertical axis we report the frequency ν(s) of IGSs versus the silhouette value *s*; this value is between −1 and +1. The average values are 0.42 for all the clusters.

**Figure A8:**
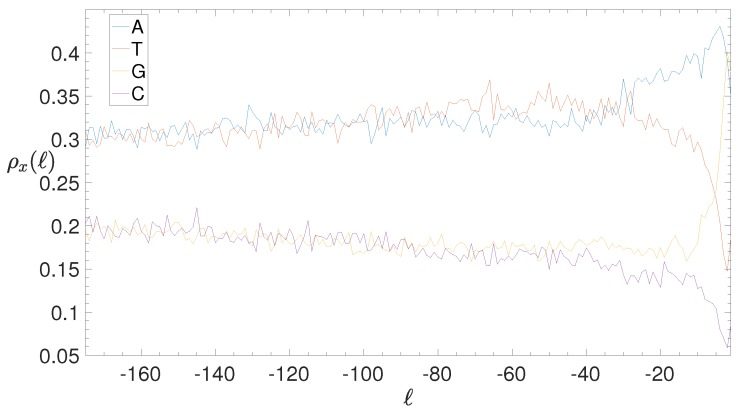
*B. subtilis*. BCA of the IGSs: on the vertical axis we report the density $\rho_{x}\left(\ell \right)$ (see Section 2.4) of each of the four nucleotides x= A (blue), T (red), G (yellow), C (purple) as a function of the position *ℓ* along the annotated 2338 IGSs.

**Figure A9:**
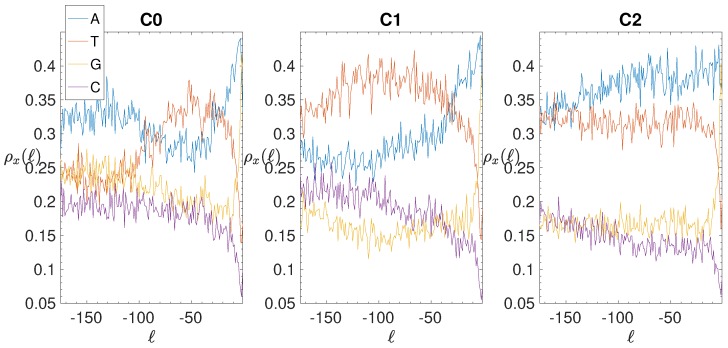
*B. subtilis*. Base composition analysis in the clusters of the IGSs: on the vertical axis we report the density $\rho_{x}\left(\ell \right)$ (see Section 2.4) of each of the four nucleotides x= A (blue), T (red), G (yellow), C (purple) as a function of the position *ℓ* along the IGS belonging to the clusters C0 (left panel), C1 (central panel) and C2 (right panel).

**Figure A10:**
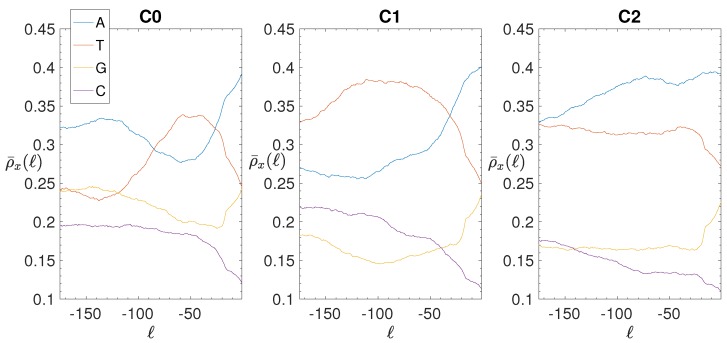
*B. subtilis*. Smoothed Base composition analysis in the clusters of the IGSs: on the vertical axis we report the averaged density $\bar{\rho }\left(\ell \right)$ for a=15 bps (see Section 2.4) of each of the four nucleotides A (blue), T(red), G (yellow), C (purple) as a function of the position *ℓ* along the IGSs belonging to the clusters C0 (left panel), C1 (central panel) and C2 (right panel).

**Figure A12:**
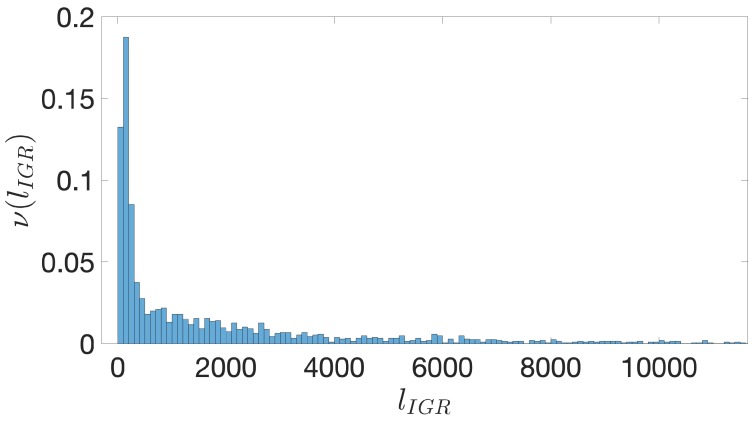
*P. haloplanktis*. The frequency $\nu (l_{IGR})$ of IGRs versus their length, $l_{IGR}$, expressed in bps. The binning is over 100 bps. The distribution is truncated at 12,000 bps.

**Figure A13:**
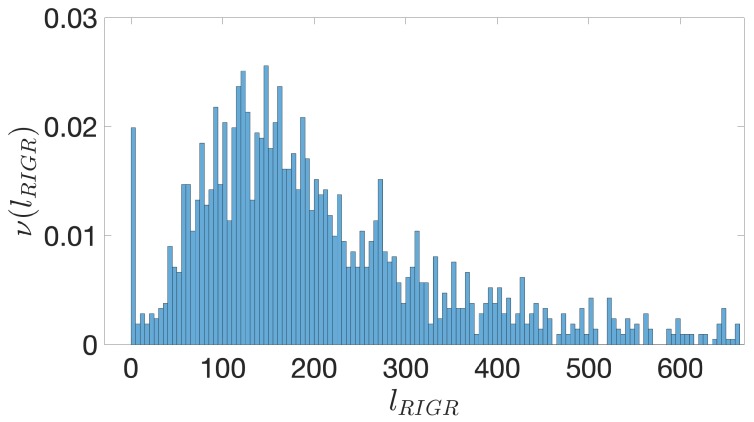
*P. haloplanktis*. The frequency $\nu (l_{RIGR})$ of RIGRs versus their length, $l_{RIGR}$, expressed in bps. The binning is over 5 bps and the distribution is truncated at 600 bps. The peak close to 0 is due to the simplifying assumption of setting to 0 the contribution from overlapping coding regions.

**Figure A14:**
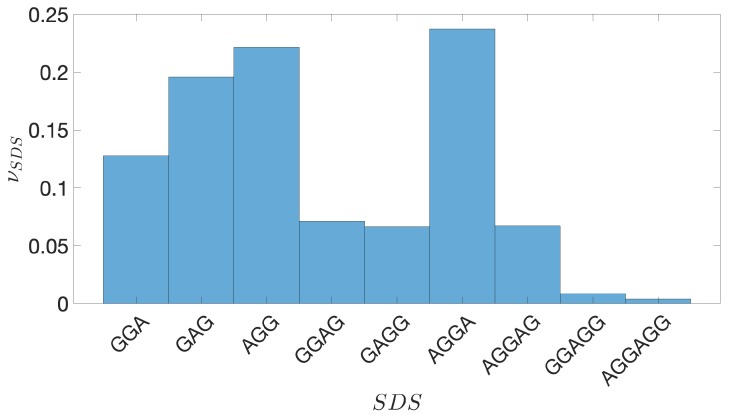
*P. haloplanktis*. The frequency $\nu_{SDS}$ of the different SDSs located upstream the TSC, listed along the horizontal axis.

**Figure A15:**
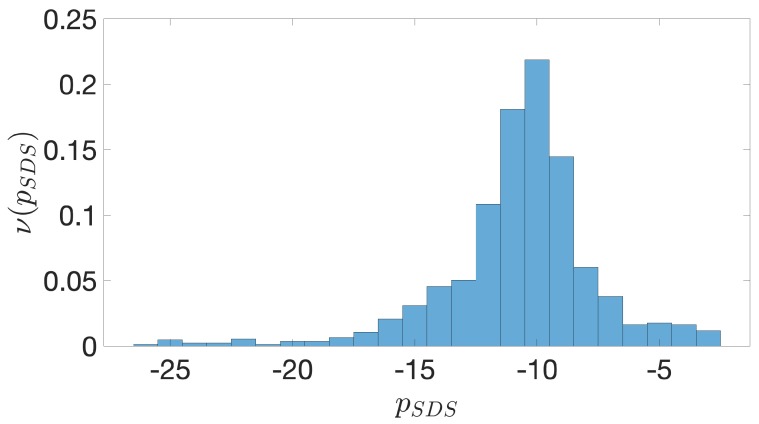
*P. haloplanktis*. The frequency $\nu (p_{SDS})$ of the position $p_{SDS}$ of the SDSs upstream the TSC.

**Figure A16:**
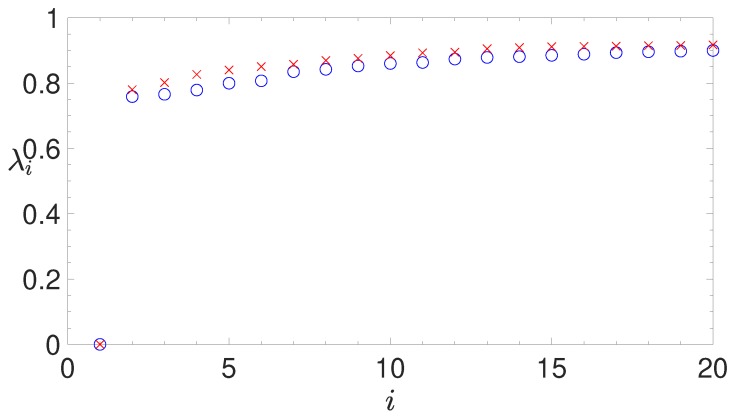
*P. haloplanktis*. The first twenty eigenvalues in ascending order of the normalized Laplacian matrix obtained by the alignment of the IGSs. Red crosses and blue circles correspond to different values of the similarity threshold, determined by the two statistical approaches described in Section 2.3.2. A better discrimination of the three main eigenvalues is obtained for a higher similarity threshold (blue crosses), which corresponds to the second statistical approach, better suited for short sequences, as the annotated IGSs.

**Figure A17:**
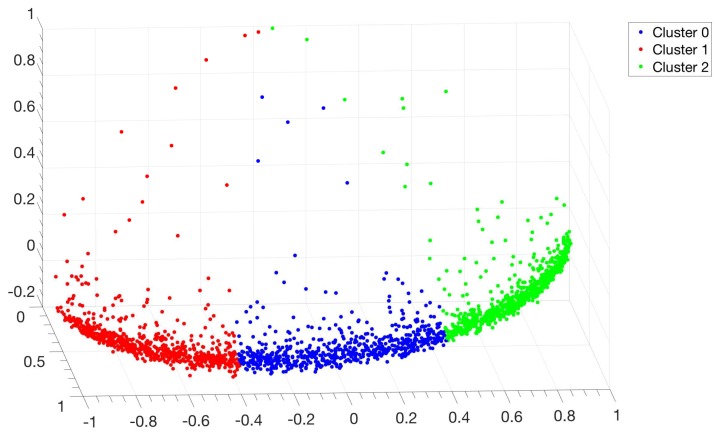
*P. haloplanktis*. Distribution of points in the clustering space relative to the alignment of the IGSs. Each point represents an IGS and the color code corresponds to the three clusters identified by the *Clustering Algorithm* described in Section 2.3.3. This seemingly unusual distribution of points in the clustering space is due to the presence of scattered points which correspond to IGSs very far from the centroid of the different clusters. This is peculiar of this bacterium; on the other hand if these few atypical IGSs would be eliminated from the sample one should recover a point distribution very similar to those reported in Figure 8 and Figure A6.

**Figure A18:**
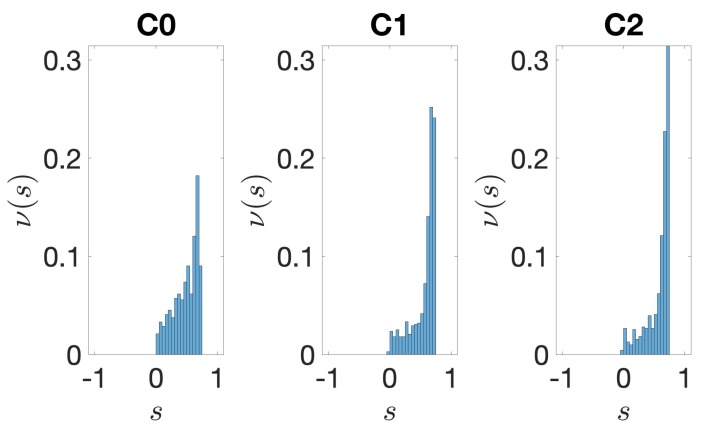
*P. haloplanktis*. Distribution of silhouette values relative to the clustering of the IGSs. On the vertical axis we report the frequency ν(s) of IGSs versus the silhouette value *s*; this value is between −1 and +1. The average values are 0.48 for cluster C0, 0.56 for C1 and 0.58 for C2.

**Figure A19:**
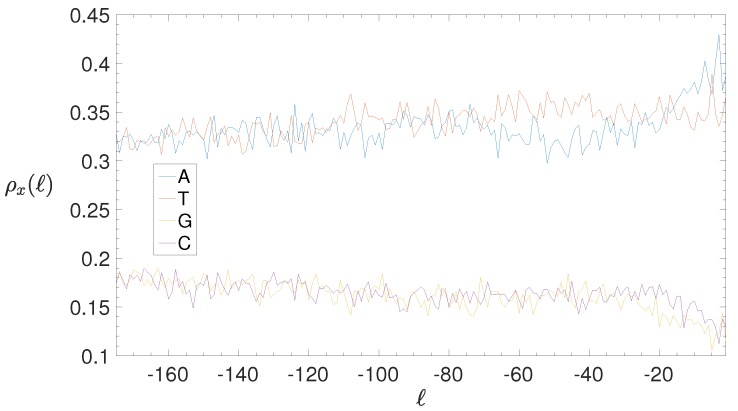
*P. haloplanktis*. BCA of the IGSs: on the vertical axis we report the density $\rho_{x}\left(\ell \right)$ (see Section 2.4) of each of the four nucleotides x= A (blue), T(red), G (yellow), C (purple) as a function of the position *ℓ* along the annotated 2091 IGSs.

**Figure A20:**
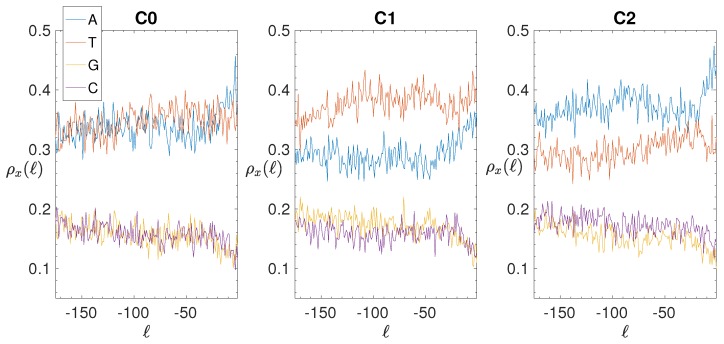
*P. haloplanktis*. Base composition analysis in the clusters of the IGSs: on the vertical axis we report the density $\rho_{x}\left(\ell \right)$ (see Section 2.4) of each of the four nucleotides x= A (blue), T(red), G (yellow), C (purple) as a function of the position *ℓ* along the IGS belonging to the clusters C0 (left panel), C1 (central panel) and C2 (right panel).

**Figure A21:**
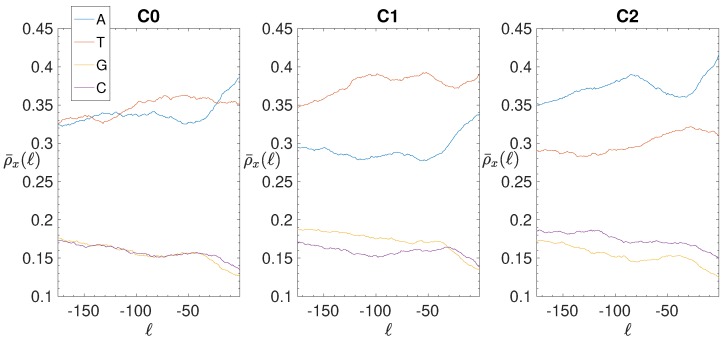
*P. haloplanktis*. Smoothed Base composition analysis in the clusters of the IGSs: on the vertical axis we report the averaged density $\bar{\rho }\left(\ell \right)$ for a=15 bps (see Section 2.4) of each of the four nucleotides A (blue), T(red), G (yellow), C (purple) as a function of the position *ℓ* along the IGSs belonging to the clusters C0 (left panel), C1 (central panel) and C2 (right panel).

## Appendix A. Supporting Information

In order to account for effectiveness of IGS analysis in bacterial species here we report the data for two other species: *B. subtilis* and *P. haloplanktis*. In fact, from Figure A1, Figure A2, Figure A3, Figure A4, Figure A5, Figure A6, Figure A7, Figure A8, Figure A9, Figure A10 and Figure A11 and Table A1 and Table A2 have been obtained for the former species, while from Figure A12, Figure A13, Figure A14, Figure A15, Figure A16, Figure A17, Figure A18, Figure A19, Figure A20, Figure A21 and Figure A22 and Table A3 and Table A4 refer to the latter one. Both sets of pictures and tables follow the same ordering adopted for *E. coli* in the main text.

First of all, we can remark that the global BCA is quite similar for the three species, although the cluster BCA exhibits peculiar features, typical of each species. This notwithstanding, the figures appearing in the table of the three bacterial species (Table 1 and Table 2 for *E. coli*, Table A1 and Table A2 for *B. subtilis*, Table A3 and Table A4 for *P. haloplanktis*) are quite similar to each other in all of the three species, thus testifying an overall robustness of the structural features in the gene networks associated to clusters.

The remarkable difference is highlighted in Figure A17: the crescent like shape of point distribution in the clustering space is due to the presence of a relative small subset of IGSs, that contain an unusually high amount of G and C nucleotides. AS a final remark it is worth pointing out that, while the conclusion drawn about the COG functional enrichment analysis provides quite similar results for *E. coli* and *B. subtilis*, one of the three clusters of *P. haloplanktis* lacks any indication of functional enrichment.

Anyway, despite the appearance of different features, we can say that the general conclusions drawn by the IGS analysis for *E. coli* apply to the two other species.

**Table A1:** Coexpression networks in *B. subtilis*. We compare the features of the coexpression networks for each cluster between the three clusters obtained by the clustering method and other three clusters obtained by averaging over a 1000 random samplings of the IGSs of *B. subtilis*.

	$N_{\mathrm{IGS}}$	$N_{\mathrm{genes}}$	$N_{\mathrm{LCC}}$	${\bar{N}}_{\mathrm{LCC}}$	$\sigma_{\mathrm{LCC}}$	$\frac{N_{\mathrm{LCC}}-{\bar{N}}_{\mathrm{LCC}}}{\sigma_{\mathrm{LCC}}}$	$N_{\mathrm{link}}$	${\bar{N}}_{\mathrm{link}}$	$\sigma_{\mathrm{link}}$	$\frac{N_{\mathrm{link}}-{\bar{N}}_{\mathrm{link}}}{\sigma_{\mathrm{link}}}$
C0	749	1341	25	25.7	14.4	−0.05	63	169.6	189.7	−0.56
C1	856	1346	41	30.6	15.6	0.67	417	199.1	201.2	1.08
C2	884	1487	16	32.3	16.5	−0.99	28	217.5	212.2	−0.89

**Table A2:** Cooccurrence networks in *B. subtilis*. We compare the features of the cooccurrence networks for each cluster between the three clusters obtained by the clustering method and other three clusters obtained by averaging over a 1000 random samplings of the IGSs of *B. subtilis*.

	$N_{\mathrm{IGS}}$	$N_{\mathrm{genes}}$	$N_{\mathrm{LCC}}$	${\bar{N}}_{\mathrm{LCC}}$	$\sigma_{\mathrm{LCC}}$	$\frac{N_{\mathrm{LCC}}-{\bar{N}}_{\mathrm{LCC}}}{\sigma_{\mathrm{LCC}}}$	$N_{\mathrm{link}}$	${\bar{N}}_{\mathrm{link}}$	$\sigma_{\mathrm{link}}$	$\frac{N_{\mathrm{link}}-{\bar{N}}_{\mathrm{link}}}{\sigma_{\mathrm{link}}}$
C0	749	1341	73	50.2	16.6	1.38	162	119.8	60.9	−0.69
C1	856	1346	71	63.1	17.4	0.45	198	158.3	77.0	0.52
C2	884	1487	38	67.4	18.4	−1.60	85	170.1	84.2	−1.01

**Table A3:** Coexpression networks in *P. haloplanktis*. We compare the features of the coexpression networks for each cluster between the three clusters obtained by the clustering method and other three clusters obtained by averaging over a 1000 random samplings of the IGSs of *P. haloplanktis*.

	$N_{\mathrm{IGS}}$	$N_{\mathrm{genes}}$	$N_{\mathrm{LCC}}$	${\bar{N}}_{\mathrm{LCC}}$	$\sigma_{\mathrm{LCC}}$	$\frac{N_{\mathrm{LCC}}-{\bar{N}}_{\mathrm{LCC}}}{\sigma_{\mathrm{LCC}}}$	$N_{\mathrm{link}}$	${\bar{N}}_{\mathrm{link}}$	$\sigma_{\mathrm{link}}$	$\frac{N_{\mathrm{link}}-{\bar{N}}_{\mathrm{link}}}{\sigma_{\mathrm{link}}}$
C0	664	1074	22	23.0	12.0	−0.08	36	102.7	108.8	−0.61
C1	718	1182	47	26.8	13.8	1.46	305	124.6	128.4	1.40
C2	709	1079	24	26.1	12.0	−0.16	42	122.1	121.6	−0.66

**Table A4:** Cooccurrence networks in *P. haloplanktis*. We compare the features of the cooccurrence networks for each cluster between the three clusters obtained by the clustering method and other three clusters obtained by averaging over a 1000 random samplings of the IGSs of *P. haloplanktis*.

	$N_{\mathrm{IGS}}$	$N_{\mathrm{genes}}$	$N_{\mathrm{LCC}}$	${\bar{N}}_{\mathrm{LCC}}$	$\sigma_{\mathrm{LCC}}$	$\frac{N_{\mathrm{LCC}}-{\bar{N}}_{\mathrm{LCC}}}{\sigma_{\mathrm{LCC}}}$	$N_{\mathrm{link}}$	${\bar{N}}_{\mathrm{link}}$	$\sigma_{\mathrm{link}}$	$\frac{N_{\mathrm{link}}-{\bar{N}}_{\mathrm{link}}}{\sigma_{\mathrm{link}}}$
C0	664	1074	58	45.3	21.2	0.60	129	104.8	71.7	0.34
C1	718	1182	89	51.4	21.5	1.75	441	120.3	77.1	4.16
C2	709	1079	36	51.8	22.5	−0.70	76	122.6	86.2	−0.54
